# Team Performance Indicators Explain Outcome during Women’s Basketball Matches at the Olympic Games

**DOI:** 10.3390/sports5040096

**Published:** 2017-12-17

**Authors:** Anthony S. Leicht, Miguel A. Gomez, Carl T. Woods

**Affiliations:** 1 Sport and Exercise Science, James Cook University, Townsville 4811, Australia; Carl.Woods@jcu.edu.au; 2 Faculty of Physical Activity and Sport Sciences, Polytechnic University of Madrid, Madrid, Spain; magor_2@yahoo.es

**Keywords:** team sports, classification tree, machine learning, performance analysis, non-linear analysis, athlete

## Abstract

The Olympic Games is the pinnacle international sporting competition with team sport coaches interested in key performance indicators to assist the development of match strategies for success. This study examined the relationship between team performance indicators and match outcome during the women’s basketball tournament at the Olympic Games. Team performance indicators were collated from all women’s basketball matches during the 2004–2016 Olympic Games (*n* = 156) and analyzed via linear (binary logistic regression) and non-linear (conditional interference (CI) classification tree) statistical techniques. The most parsimonious linear model retained “defensive rebounds”, “field-goal percentage”, “offensive rebounds”, “fouls”, “steals”, and “turnovers” with a classification accuracy of 85.6%. The CI classification tree retained four performance indicators with a classification accuracy of 86.2%. The combination of “field-goal percentage”, “defensive rebounds”, “steals”, and “turnovers” provided the greatest probability of winning (91.1%), while a combination of “field-goal percentage”, “steals”, and “turnovers” provided the greatest probability of losing (96.7%). Shooting proficiency and defensive actions were identified as key team performance indicators for Olympic female basketball success. The development of key defensive strategies and/or the selection of athletes highly proficient in defensive actions may strengthen Olympic match success. Incorporation of non-linear analyses may provide teams with superior/practical approaches for elite sporting success.

## 1. Introduction

Basketball is the second most popular team sport worldwide, and the second most watched Olympic sport, with over 450 million registered players [1]. The key physical and physiological characteristics of basketball athletes have been documented [2,3,4] and reported to contribute to individual performance [5,6] with team success reliant on the coherent integration of individual performances [7,8]. Many studies have examined the importance of team performance indicators for match success within national junior and senior competitions [9,10,11,12,13]. Most have identified “field-goal percentage”, “defensive rebounds”, and “assists” as crucial team indicators for match success [9,11,12,13,14]. Recently, these results were extended to the elite international level with “field goal percentage” and “defensive rebounds” identified as vital for match outcomes at the men’s Olympic basketball tournaments [8]. Key team performance was identified using both linear and non-linear statistical techniques with a classification tree (a non-linear machine learning technique) providing coaches with a practical guide to inform match strategy and team selection [8]. Recently, others have utilized a classification and regression tree to identify predictors of winning within the Spanish EBA Basketball League [15]. Similar analytical approaches have been applied in elite Australian Football [16] and rugby league [17] and highlight these novel techniques as important tools for sport scientists and coaches to improve on-field success. However, most studies examining team performance indicators and match success have focused on male athletes [9,10,11,12,13,14,16,17] with very few studies examining females [18,19,20], and less for basketball [18,19,20]. Performance differences due to dissimilarities in anthropometrical and fitness characteristics between male and female basketball athletes [21] may impact on game-related statistics and match success [18]. Subsequently, it is critical to examine predictors of match success for elite female basketball athletes for a greater understanding of factors contributing to match success. 

To date, only two studies have focused on female basketball athletes and match performance indicators for success. Gomez et al. [19] examined matches within the 2004/2005 women’s Spanish professional league and demonstrated that “1-point” and “3-point field-goal percentage”, “assists”, and “defensive rebounds” were important during balanced games (score-differences ≤12 points) and “2-point field-goal percentages”, “defensive rebounds”, and “steals” during unbalanced games (score-differences >12 points). In a second study, Gomez et al. [7] examined the impact of starter/nonstarter player status, team performance indicators, and match outcome within the 2005 Women’s National Basketball Association. These authors reported that shooting (2-point field-goals, successful free-throws) and passing capability (assists) were discriminatory of player status with this profile impacting on match success. Recently, others examined match performance in elite, female Spanish basketball with steals and assists correlated with a range of physical fitness characteristics (e.g., speed, agility, anaerobic power, repeated sprint ability, and aerobic power) [6]. To our knowledge, no other studies have examined match outcome and team performance indicators for female basketball players. Identification of the relationship between team performance indicators and match success, particularly at the elite level, would provide significant guidance to coaches and athletes in the development of training and match strategies for match success.

The aim of the current study was to identify the relationship between team performance indicators and match outcome during elite women’s basketball competition using linear and non-linear statistical techniques. Based on previous results [8], it was hypothesized that distinctive performance indicator combinations would explain match outcome with the non-linear technique, offering greater practical utility for coaches and athletes.

## 2. Materials and Methods

This study was a retrospective analysis of publically available data from the official Olympic websites. All matches (*n* = 156) undertaken within the women’s basketball tournament at the past four Olympic tournaments (2016, Rio de Janeiro, *n* = 38; 2012, London, *n* = 38; 2008, Beijing, *n* = 38; 2004, Athens, *n* = 42) were examined. As previously described [8], team performance indicators (‘field-goal percentage”, “3-point percentage”, “2-point percentage”, “free-throw percentage”, “offensive rebounds”, “defensive rebounds”, “assists”, “turnovers”, “steals”, “blocked shots”, “fouls committed”, and “fouls against”) were downloaded, collated, and *a priori* classified according to match outcome (win/loss). Normalization of all team performance indicators was undertaken using the number of ball possessions, as previously described [8,13,22]. Two datasets (one per team) were obtained from each match with 312 datasets (76 from 2016, 76 from 2012, 76 from 2008, 84 from 2004) examined in the current study.

Relative to match outcome, descriptive statistics (mean ± SD) were calculated for each team performance indicator with all analyses and visualizations conducted using R (version 3.2.2, Vienna, Austria). Match outcome comparisons of each performance indicator were examined via multivariate analysis of variance (MANOVA) with the level of statistical significance set at *p* < 0.05. The magnitude of effect (i.e., effect size and 90% confidence intervals) for match outcome comparisons were calculated using Cohen’s *d* statistic as follows: *d* < 0.2: trivial; *d* = 0.20–0.49: small; *d* = 0.50–0.79: medium; *d* > 0.79: large [23].

As described previously [8], data were examined via both binary logistic regression and a conditional interference (CI) classification tree. Briefly, match outcome was coded as the response variable with each identified performance indicator coded as the explanatory variable within both statistical techniques. Model parsimony for the binary logistic regression was performed using the delta Akaike Information Criterion (AIC) and Akaike weights [24] via the “dredge” function in the MuMIn package [24]. A null model was built and used as a comparator. A recursively, partitioned CI, classification tree was grown via the “ctree” function in the party package [25] with a minimum node size of 5 observations chosen for partitioning. This type of classification tree was chosen as its fitting algorithm corrects for multiple testing, thus avoiding overfitting [25]. Accordingly, this analysis results in the growth of an unbiased decision tree that does not require pruning [25].

## 3. Results

During wins, all of the normalized, team performance indicators were significantly greater than for losses, with the exception for “turnovers”, which was significantly lower, and “fouls committed”, which was similar (Table 1). The indicators that had the largest effect on match outcome were “field-goal percentage”, “defensive rebounds”, “assists”, and “steals” (Table 1).

The following performance indicators were retained by the best linear model: “defensive rebounds”, “field-goal percentage”, “offensive rebounds”, “fouls”, “steals”, and “turnovers” (Table 2). This model successfully identified 88.5% and 89.1% of the *a priori* classified wins and losses, respectively, for an average model accuracy of 85.6%.

**Figure 1:**
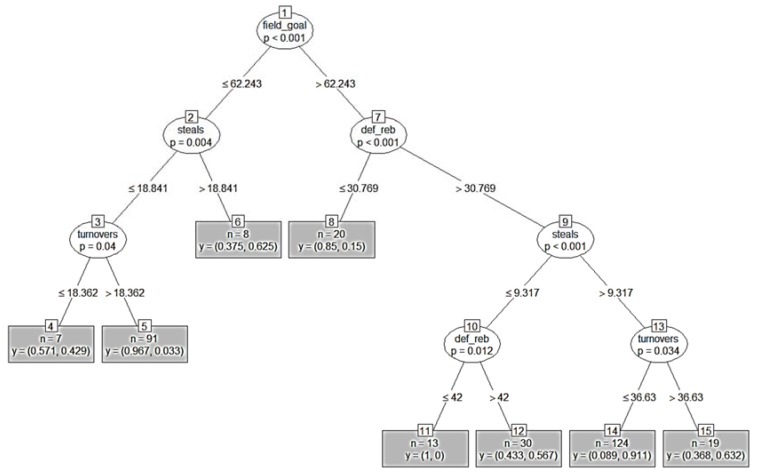
The conditional interference classification tree highlighting the probability of wins and losses during the women’s basketball tournament of the 2004–2016 Olympic Games. “*n*” denotes the number of observations or datasets in each node (minimum of 5) with the first y-value denoting the probability of losing and the second y-value denoting the probability of winning (e.g., 0.7 = 70%). field_goal = “field-goal percentage”; def_reb = “defensive rebounds”; values for each team performance indicators were normalized to ball possessions.

Four performance indicators were retained within the CI classification tree (Figure 1) with the tree successfully classifying 94.2% and 78.2% of the *a priori* classified wins and losses, respectively, for an average model accuracy of 86.2%. The root node (Number 1) partitioned the dataset based on “field-goal percentage” and generated eight terminal nodes (Numbers 4, 5, 6, 8, 11, 12, 14, and 15). The left-hand branching of the tree denoted primarily a loss (field-goal percentage ≤62.243), while the branching to the right primarily denoted a win (field-goal percentage >62.243). 

On the left-hand side of the tree, Node Number 2 separated the data based on “steals” and generated Terminal Node 6, while Node Number 3 further separated the data based on “turnovers” to generate Terminal Nodes 4 and 5. The combination of “field-goal percentage” (≤62.243%), “steals” (≤18.841), and “turnovers” (>18.362) provided the greatest probability of losing (96.7%, Terminal Node 5).

On the right-hand side of the tree, Node Number 7 separated the data based on “defensive rebounds” and generated Terminal Node 8, while Node Number 9 further separated the data based on “steals” to generate Nodes 10 and 13. Finally, Nodes 10 and 13 split the data based upon by “defensive rebounds” and “turnovers,” respectively. The combination of “field-goal percentage” (>62.243%), “defensive rebounds” (>30.789), “steals” (>9.317), and “turnovers” (<36.63) provided the greatest probability of winning (91.1%, Terminal Node 14).

## 4. Discussion

The current study identified the key team performance indicators that contributed to success in women’s basketball at the 2004–2016 Olympic Games. The non–linear analysis resolved a combination of “field-goal percentage”, “defensive rebounds”, “steals”, and “turnovers” as providing the greatest probability of winning (91.1%). Further, a unique combination of “field-goal percentage”, “steals”, and “turnovers” offered the lowest probability of winning (3.3%) and the greatest probability of losing (96.7%). Overall, the average model accuracy was marginally higher for the CI classification tree compared with the logistic regression analysis and likely provided coaches and analysts with a flexible model to manipulate game plans or strategies to enhance the likelihood of winning. The use of non-linear, machine learning techniques may provide sport scientists with greater support when assisting coaches with decisions regarding match strategy design, team selection or identifying opponent strengths and weaknesses [8,15].

In our previous work, “field-goal percentage”, “defensive rebounds”, “steals”, and “turnovers” were identified as key indicators of outcome for men’s matches at the Olympic Games [8]. The current results extend these findings to women’s matches at the Olympic Games and confirm these indicators as significant, sex-independent contributors to basketball success at the current Olympic level. Further, our results for Olympic matches highlight shooting proficiency and defensive actions as vital for success in both men’s and women’s basketball. Others have reported the importance of “field-goal percentage” [7,9,13,14,20,26], “defensive rebounds” [9,10,12,13,14,15,20,26] and “turnovers” [13,14] for basketball match success in various competitions. While shooting capability may seem apparent for match success, particularly longer distance shots for females [19], collectively the current and prior results [9,10,12,13,14,20,26] confirmed defensive actions as critical for match success. Gomez et al. [9] identified “defensive rebounds” as the predominant performance indicator to discriminate winning and losing within the Spanish Men’s Basketball League. Similarly, Trninic et al. [14] identified “defensive rebounds” as the key discriminator for success at the European club championships. These authors commented that winning teams exhibited a greater discipline and balance of play highlighted by greater decision making and teamwork [14]. Subsequently, inclusion of athletes that are familiar with each other and a controlled style of play [15], or who are more tactically disciplined [22], may provide greater defensive actions for match success. This degree of familiarity, focus, and discipline may be difficult given the limited preparation time and match opportunities for national teams that include athletes competing potentially in every corner of the world. Therefore, preparatory activities for individual athletes, development of team cohesion and a focus on team defensive activities may be vital for Olympic success. Increasing defensive pressure on the opposition was reported to reduce basketball athlete’s preference to shoot [27] that may provide further impact on shooting proficiency and overall match success. Coaches are encouraged to develop key defensive strategies and/or selection of athletes highly proficient in defensive actions for greater Olympic match success.

A key finding of the current study was the substantial effect of “steals” on match success. This result extended our previous finding that “steals” was a key performance indicator for elite Olympic basketball success [8] and unbalanced games within the Spanish Women’s League [19], and confirms “steals” as an important focus area for coaches and athletes. Interestingly, “steals” were reported to differentiate men’s and women’s teams within an analysis of close matches during the basketball World Championships in 1999–2002 [18]. Compared to women’s teams, men’s teams were associated with a lower proportion of “steals” which was related to their anthropometric characteristics (i.e., taller and heavier) [18]. Our current and previous [8] results indicate similar sex differences for “steals” during wins (women’s = 15.5 vs. men’s = 10.5) but highlight further the importance of this indicator for match success, independent of sex. Subsequently, coaches of men’s and women’s teams are encouraged to develop strategies to enhance “steals” during elite basketball matches. These strategies may include full- and half-court presses and double teaming of players to enhance the likelihood of match success [22,28]. Further, selection of athletes that possess superior fitness characteristics may be important for the generation of “steals.” Previously, “steals” were associated with superior speed, agility, anaerobic power, and repeated sprint ability in elite, junior, female basketball athletes [6]. The inclusion of athletes that possess these characteristics for national teams may provide the talent base to enhance “steals” during matches and ultimate success. Additionally, development of these fitness characteristics, specific to each position [5], during the pre-Olympic period may be suggested as a priority for coaches in their preparation for the games [29]. 

The current study has expanded the understanding of match success for Olympic women’s basketball competition. Through the use of linear and non-linear statistical techniques, key performance indicators were identified to assist coaches and athletes in their preparation for international, women’s basketball competition. However, some limitations of the current study should be discussed. Firstly, only matches of the most recent Olympic Games were examined with future analyses needed to examine the robustness of the current models for match success within major international competitions including the Olympic Games. Additionally, matches within all rounds of the competition (regular and playoff) were examined within the current analyses. While a previous study indicated varying match success reliance on team performance indicators within different stages of seasonal competition [26], we expected this to be of little impact for a short-term tournament like the Olympic Games where each success had a substantial impact on final tournament success. Furthermore, analyses were conducted without examination of the impact of prior matches. Previously, accumulated and moderate fatigue from consecutive matches during a Spanish Basketball Federation tournament was suggested to impact three-point shooting accuracy and/or defensive actions [12]. Future examination of the impact of consecutive matches on team performance indicators and match success may clarify the role of fatigue and relevance of physical conditioning for Olympic success. Further, examination of athlete workloads during matches, possibly via wearable technology, in conjunction with team match performance indicators may identify successful team profiles to assist coaches with strategic planning during elite basketball competition.

## 5. Conclusions

The current study has identified shooting proficiency and defensive actions (e.g., “defensive rebounds”, “steals”) as quintessential for match success during a women’s Olympic basketball tournament. The unique combination of these performance indicators can provide coaches with a greater probability of winning elite matches (>91%). The development of key defensive strategies and/or the selection of athletes highly proficient in, and/or possessing fitness characteristics conducive to, defensive actions may strengthen Olympic match success. The use of non-linear, analytical techniques may provide sport scientists and coaches with superior and practical approaches to exploring multivariate datasets in elite sports for elite success.

## Figures and Tables

**Table 1:** Descriptive statistics for each team performance indicator relative to match outcome. Values are mean ± SD with each normalized to ball possessions.

Performance Indicator	Wins	Losses	*d* (90% CI)	Interpretation
Field-goal percentage	77.9 ± 13.8	60.6 ± 12.8 *	1.30 (1.09, 1.50)	Large
Free-throw percentage	129.4 ± 22.1	117.6 ± 23.0 *	0.52 (0.33, 0.71)	Medium
Offensive rebounds	22.2 ± 8.4	17.4 ± 8.7 *	0.55 (0.36, 0.74)	Medium
Defensive rebounds	47.4 ± 9.7	35.9 ± 9.2 *	1.21 (1.00, 1.41)	Large
Assists	27.9 ± 10.4	19.2 ± 8.5 *	0.91 (0.71, 1.10)	Large
Turnovers	25.7 ± 8.0	28.4 ± 7.5 *	−0.35 (−0.54, −0.16)	Small
Steals	15.5 ± 5.4	10.8 ± 5.1 *	0.90 (0.71, 1.10)	Large
Blocked shots	5.7 ± 3.8	3.4 ± 2.9 *	0.66 (0.47, 0.85)	Medium
Fouls committed	30.4 ± 8.5	31.4 ± 8.2	−0.13 (−0.32, 0.06)	Small
Fouls against	33.2 ± 10.1	29.3 ± 9.0 *	0.41 (0.23, 0.60)	Small

*n* = 312; * *p* < 0.005 vs. Wins; *d*—effect size; CI—confidence interval.

**Table 2:** Model summary for the binary logistic regression analysis ranked according to the delta Akaike Information Criterion and Akaike weights.

Predictors	*LL*	*df*	AICc	ΔAIC	*w*_i_
~def_reb + field_goal + off_reb + fouls + steals + turnovers	−82.93	7	180.23	<0.01	0.15
~blocked_shots + def_reb + field_goal + fouls + off_reb + steals + turnovers	−82.50	8	181.47	1.24	0.08
~ def_reb + field_goal + fouls + steals + turnovers	−84.68	6	181.63	1.40	0.07
~def_reb + field_goal + fouls + free_throw + off_reb + steals + turnovers	−82.72	8	181.93	1.70	0.06
~assists + def_reb + field_goal + fouls + off_reb + steals + turnovers	−82.88	8	182.24	2.01	0.05
~def_reb + field_goal + fouls + fouls_against + off_reb + steals + turnovers	−82.92	8	182.31	2.08	0.05
~blocked_shots + def_reb + field_goal + fouls + steals + turnovers	−84.03	7	182.43	2.20	0.05
~blocked_shots + def_reb + field_goal + fouls + free_throw + off_reb + steals + turnovers	−82.27	9	183.13	2.90	0.04
Null (~1)	−216.26	1	434.54	254.31	<0.01

*LL*: log likelihood; *df*: degrees of freedom; AICc: Akaike Information Criterion; ΔAIC: delta AIC; *w*_i_: Akaike weight; def_reb: defensive rebounds; field_goal: field goal percentage; off_reb: offensive rebounds; free_throw: free-throw percentage.
